# Animals Exposed to *Leptospira* Serogroups Not Included in Bacterins in the United States and Puerto Rico

**DOI:** 10.3390/tropicalmed8030183

**Published:** 2023-03-22

**Authors:** Tammy Anderson, Camila Hamond, Andréa Haluch, Kari Toot, Jarlath E. Nally, Karen LeCount, Linda K. Schlater

**Affiliations:** 1 National Veterinary Services Laboratories, APHIS, U.S. Department of Agriculture, Ames, IA 50010, USA; 2 Infectious Bacterial Diseases Research Unit, ARS, U.S. Department of Agriculture, Ames, IA 50010, USA

**Keywords:** *Leptospira*, animals, MAT, United States, Puerto Rico

## Abstract

Leptospirosis is a worldwide zoonotic disease. Pathogenic leptospires colonize the renal tubules and genital tract of animals and are excreted via urine. Transmission occurs via direct contact or through contaminated water or soil. The microscopic agglutination test (MAT) is the gold standard for the serodiagnosis of leptospirosis. The present study aims to evaluate animal exposure to *Leptospira* in the U.S. and Puerto Rico during the period 2018–2020. The presence of antibodies against pathogenic *Leptospira* spp. was assessed with the MAT according to the standards of the World Organisation for Animal Health. A total of 568 sera were submitted for diagnostic, surveillance, or import/export testing from the U.S. and Puerto Rico. Seropositivity (≥1:100) was 51.8% (294/568) with agglutinating antibodies found in 115 (39.1%) cattle, 84 (28.6%) exotic animals, 38 (12.9%) horses, 22 (7.5%) goats, 15 (5.1%) dogs, 11 (3.7%) swine, and 9 (3.1%) sheep. The most detected serogroups were Australis, Grippotyphosa, and Ballum. The results showed that animals were exposed to serogroups/serovars not included in commercial bacterins such as Ballum, Bratislava (only in swine vaccine), and Tarassovi. Our findings suggest that more studies should include culture and concomitant genotyping to reduce animal disease and zoonotic risk through efficacious vaccine and diagnostic strategies.

## 1. Introduction

Leptospirosis is a zoonotic disease caused by pathogenic bacteria of the genus *Leptospira*. This zoonotic disease is endemic in least-developed and developing countries, and it is considered emerging or re-emerging in developed countries [1,2]. Leptospires survive in the renal tubules of chronically infected maintenance hosts without any obvious clinical symptoms and are shed into the environment in urine. Leptospires can survive for long periods of time in a range of environmental conditions, including soil and water, thus increasing the probability of infecting a susceptible host [3]. Humans contract the disease through contact with the urine of infected animals or environmental sources, such as lakes and rivers, contaminated with *Leptospira* [4]. In the United States, where the disease is rarely recognized [4,5], infections have been associated with occupational exposure to infected animals [6,7], recreational exposures such as swimming [8], and individuals living in economically disadvantaged urban inner-city environments [4,5].

Many species of wild and domestic animals may serve as carriers or maintenance hosts for *Leptospira* spp., in which most leptospiral infections are subclinical. However, infections may cause heavy economic losses due to abortion, stillbirth, and reduced milk production [9]. Leptospirosis can cause severe clinical disease in dogs, such as acute hepatic and/or renal failure. It can also produce a chronic carrier status, presenting as idiopathic polyuria/polydipsia that may not be preceded by severe hepatic or renal disease [10]. The epidemiology of animal leptospirosis varies by geographic area and changes over time in relation to the spread of maintenance hosts [11].

Studies conducted in the U.S. and Puerto Rico indicated that cattle were exposed to multiple serogroups of *Leptospira*, including Sejroe, Pomona, Icterohaemorrhagiae, Canicola, Grippotyphosa, Pyrogenes, Ballum, Autumnalis, Tarassovi, and Australis [12,13]. Horses were exposed to Pomona, Grippotyphosa, Australis, and Sejroe [14,15,16]. Swine were exposed to Australis [17,18]. Dogs were exposed to Autumnalis, Grippotyphosa, Australis, Pomona, Canicola, Icterohaemorrhagiae, and Sejroe [19,20], and exotic animals were exposed to Australis, Grippotyphosa, Sejroe, Autumnalis, Pomona, Canicola, and Icterohaemorrgiae [21,22,23,24,25]. The present study aims to evaluate *Leptospira* exposure in animals from the U.S. and Puerto Rico using the microscopic agglutination test (MAT) during the period 2018–2020.

## 2. Materials and Methods

The serum samples used in this study were received at the National Veterinary Services Laboratories (Ames, IA, USA) during the period from 2018 to 2020 for leptospirosis diagnostic, surveillance, or export testing. The samples came from a variety of sources including cattle, horses, swine, sheep, goats, dogs, and exotic zoo animals, such as elephants, onagers, maned wolves, zebras, bears, hyenas, lions, oryx, cheetahs, otters, wildebeests, pandas, dolphins, ocelots, foxes, dingoes, sitatungas, bison, giraffes, elands, lynx, kudus, and okapi. The vaccination and clinical history of the animals was unavailable. In total, 568 sera were received during the period from 2018 to 2020 from 23 states, the District of Columbia, and Puerto Rico (Table S1).

The MAT was performed using a panel of 15 antigens representing 13 serogroups (Table S2) following the method previously described. A titer was considered positive at ≥1:100 [26].

## 3. Results

Of the 568 samples tested, 294 (51.8%) were seropositive with agglutinating antibodies found in 115 (39.1%) cattle, 84 (28.6%) exotic animals, 38 (12.9%) horses, 22 (7.5%) goats, 15 (5.1%) dogs, 11 (3.7%) swine, and 9 (3.1%) sheep. Seventy-six (25.9%) of these 294 samples presented with high titer reactions to two or more serogroups (Table S1).

Australis was the serogroup with the highest titer reactions (47/218) with titers ranging from 1:100 to 1:1600. Hebdomadis and Tarassovi had the lowest number of highest titer reactions (2/218). Bataviae had no single highest titer reactions. There were 76 serum samples that had more than one serogroup react as the highest titer (Table 1).

**Figure 1:**
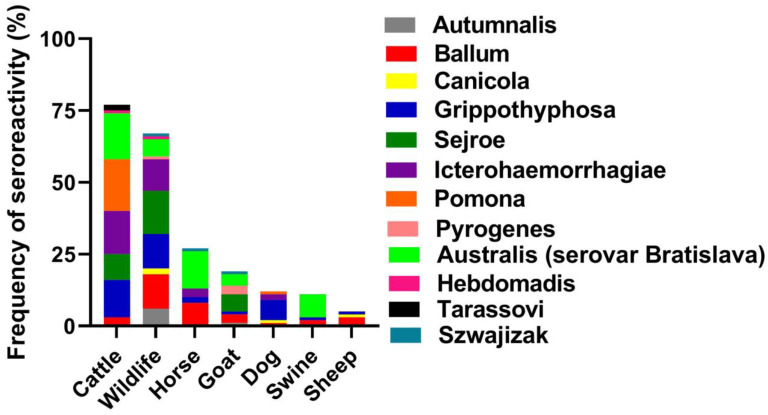
Frequency of seroreactivity of the highest titer by serogroup in 218 animals. This graph does not include negative samples or samples with more than serogroup with the highest titer.

The frequency of seroreactivity varied among species. Cattle sera showed the most reactions to Australis, Pomona, Icterohaemorrhagiae, and Grippotyphosa. Wildlife sera had the most reactivity to Sejroe. Horse and swine sera both reacted most often with Australis, while dog sera reacted most often with Grippotyphosa (Figure 1).

**Figure 2:**
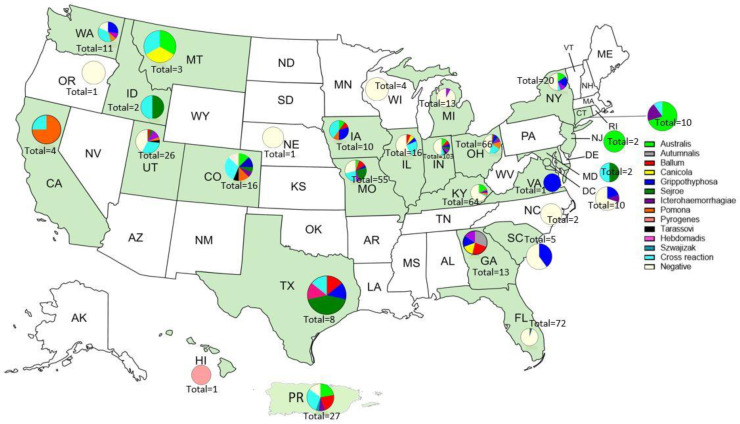
Distribution of serogroups in 23 states, the District of Columbia, and Puerto Rico in green tested using MAT.

The distribution of serogroups in the 23 states with positive samples varied. HI, NJ, and VA were the only states with sera that reacted to just one serogroup. Only one serum sample was received from both VA and HI. The serum from VA reacted to Grippotyphosa and the serum from HI reacted to Pyrogenes. Two sera samples were received from NJ and they both had the highest reaction to serogroup Australis. Sera from four states were negative (OR, NE, WI, NC); however, only one serum sample was received from both OR and NE, two from NC, and four from WI. We received the most sera from IN (103). Almost half (50) were negative and the most reactive serogroup in IN was Australis (12). Thirteen sera samples were received from MI, but only one serum sample had a reaction (Icterohaemorrhagiae). Of the five sera received from SC, three were negative and the remaining two both reacted to Grippotyphosa. The reactions in all the other states were to two or more serogroups (Table S1 and Figure 2).

## 4. Discussion

The microscopic agglutination test is the gold standard for the serodiagnosis of leptospirosis. The MAT identifies animals exposed to *Leptospira*. There are multiple disadvantages to the MAT: (a) it does not differentiate between an antibody response from natural infection and one from a vaccine response, (b) it can produce false negative results early in acute disease, (c) paired serum samples are required for the diagnosis of acute disease, (d) infected animals may be seronegative while shedding leptospires, (e) false negative results may be due to a lack of serologic cross reactivity, and (f) the MAT is laborious, subjective, and requires high levels of technical skill [9]. One way the MAT could be improved is by making sure that relevant serogroups are included in the test. The test is only as good as the data generated to support it. It is imperative to include the correct serogroups within the MAT or the results may not match the potential risk of the area.

The findings in this study revealed that animals in the U.S. are exposed to multiple serogroups of *Leptospira* spp. with epidemiological and diagnostic significance. The observed occurrence of anti-*Leptospira* agglutinins (51.7%) was not unexpected, since seroreactivity for leptospirosis is common and has been reported in the U.S. [12,13,15,16,18,19,20,22,24] and Puerto Rico [27,28,29]. The distribution around the U.S. and Puerto Rico in this study relied on the needs of the submitters for purposes such as for import/export, surveillance, or diagnostic of a suspect leptospirosis case. Therefore, we cannot make any statistical conclusions in terms of prevalence since these samples were all submitted with a specific need and not through random sampling. There could be a lot of factors that influenced the distribution of serogroups that we could not explore in this study, such as humidity, temperature, variety of hosts, etc. What is important to note from these data, however, is that we did see the serogroups indicated in Figure 2 in those states. It would be wise to consider this when choosing diagnostic testing/MAT panels or vaccines.

One limitation of the MAT is that it is unable to differentiate between antibody titers produced following vaccination and antibody responses due to natural exposure [30], and this should be considered when evaluating the significance of antibody titers. The Bratislava serovar is included in swine leptospirosis bacterins in the U.S., so it is unknown if the antibody titers detected in swine in this study were produced following vaccination or natural exposure. However, Bratislava is not included in the cattle leptospirosis bacterins. Nevertheless, our data reveal a wide variety of serogroups reacting in the MAT. In this study, the Australis serogroup (serovar Bratislava) was frequently detected in cattle, exotic animals, goats, and swine. This is consistent with previous studies that showed Bratislava antibody titers in U.S. cattle [12,13], exotic animals [25], horses [14,16], and swine [17,18]. Bratislava is a host-adapted serovar of horses in Europe [31] and swine in the U.S. [17,18,32], and is rarely isolated in the U.S. [13]. All titers to the Australis serogroup (including serovar Australis and serovar Bratislava) in cattle were 100 and 200. Without additional information on animal history, it is difficult to draw a conclusion about exposure versus vaccination in cattle. The bacterin for horses only includes serovar Pomona in the U.S. because only Pomona has been isolated from an aborted equine fetus [33]. In these data, there were titers to the Australis serogroup in horses of 1600, 800, and 400, and multiple titers of 100/200. We assume the titers are not from bacterins, since serovars from the serogroup Australis are not included in the equine bacterins; therefore, we concluded that exposure to the *Leptospira* serogroup Australis occurred. Canine bacterins historically contained only Canicola and Icterohaemorrhagiae. More recently, products including four serovars (Canicola, Icterohaemorrhagiae, Pomona, and Grippotyphosa) have become available. In Europe, the canine vaccine includes Bratislava due to epidemiological studies that showed consistent circulation and clinical cases associated with serovar Bratislava [11,34,35,36]. However, our data from the U.S., where serovar Bratislava is not included, indicated that exposure likely occurred.

Grippotyphosa was the second most frequently detected (17%) serogroup with seroreactivity in all animal sources in this study. Antibodies to serogroup Grippotyphosa were previously reported in wildlife in the U.S. [25]. Wildlife are considered an important reservoir host of serovar Grippotyphosa [9]. Serovar Grippotyphosa (serogroup Grippotyphosa) is included in leptospirosis bacterins in the U.S.; therefore, many antibody titers detected in cattle, small ruminants, dogs, or swine could be attributed to vaccination. However, there was one outstanding 25600 titer to Grippotyphosa that was associated with acute disease in an exotic animal [37]. This animal also had low titers to Icterohaemorrhagiae and Autumnalis (400 and 200, respectively). This most likely suggests that the animal was exposed to the *Leptospira* serogroup Grippotyphosa and cross reacted to Icterohaemorrhagiae and Autumnalis.

All the animal groups in this study showed seroreactivity to Ballum. A seroprevalence study showed that cattle have been exposed to serogroup Ballum in the U.S. [12]. Ballum is emerging as an important problem in humans and animals in New Zealand [38]. The *L. borgpetersenii* serogroup Ballum has been isolated from rodents, and it is virulent in the hamster model, which emulates the pathology associated with acute lethal forms of human leptospirosis [28,39]. The highest titer to Ballum seen in these data was 1600 in an exotic animal, as well as a cow and horse each with titers of 800. Ballum is not in any of the available bacterins, and it is not commonly included in many MAT panels. The data here as well as in previous studies show it is a serogroup of concern [38].

Seroreactivity against serogroup Icterohaemorrhagiae has been reported in studies conducted in U.S. cattle [12], horses [16], dogs [19,20], and wildlife [21,22,23,24,25]. Icterohaemorrhagiae is included in leptospirosis bacterins in the U.S. Rodents are the most common reservoir of Icterohaemorrhagiae, which is transmitted by direct or indirect contact with the urine of rodents [9]. With Icterohaemorrhagiae being included in many vaccines, it is hard to make conclusions on natural exposure in vaccinated animals. However, our data did show several exotic species with high titers to Icterohaemorrhagiae (≥1600).

Seroreactivity in this study was also seen for Autumnalis in exotic animals and goats, Hebdomadis in cattle and exotic animals, and Szwajizak in exotic animals, horses, and goats. Studies have shown that these serogroups have low frequency in animals around the world [9]. Only cattle presented seroreactivity to Tarassovi. Recently, the *L. borgpetersenii* serovar Tarassovi was isolated from an asymptomatic cow in the U.S. [40]. Tarassovi is an emerging serovar associated with disease in dairy cattle and is a public health risk in New Zealand [41]. None of these serogroups are included in any of the available U.S. bacterins. Hebdomadis, Szwajizak, and Tarassovi are not often included in the MAT.

Exposure is not indicative of infection, but given that current leptospirosis bacterins in the U.S. do not contain the serovars Bratislava (only in swine), Ballum, Autumnalis, Hebdomadis, Szwajizak, and Tarassovi, this finding should be explored further [13]. There is also the question of exposure to saprophytic species, especially considering the recent identification of new saprophytic species and the limited data on the reactivity of these species. Investigations should be conducted to determine whether these species could cross react in the MAT with the pathogenic species.

Recently, we used the serogroup Djasiman in our MAT panel to test four samples from horses with clinical symptoms of leptospirosis. These samples were reactive to Djasiman with titers ranging from 200 to 1600, highlighting the importance of including various relevant serogroups of *Leptospira* spp. in MAT panels. This presents an example of why leptospirosis infections are rarely recognized in the U.S.; to identify disease or exposure, the correct serogroups must be considered. Therefore, more prevalence studies are needed to determine which serovars to include in MAT panels. The MAT panel for each animal species could be different based on what the expected exposures are. It is worth considering that the use of MAT panels based on species is more appropriate and cost-effective than a generic panel for all species. If prevalence studies could be carried out for various species in the U.S., the serogroups used in the MAT could be narrowed down or expanded to match the potential exposure. For example, the National Veterinary Services Laboratories (NVSL) offers a 15-serovar panel, while many other laboratories only offer 8 serovars or fewer. Most laboratories do not include the serovars Ballum or Tarassovi; however, our data and several recently published studies have shown isolates from both of these serogroups [39,40]. Our data show that some serogroups should be added to the commonly tested panel (Ballum and Tarassovi), but also show that some may not need to be included (Bataviae). The NVSL includes Bataviae in their 15-way panel, but our data herein show that the serogroup Bataviae was never the single highest-reacting serogroup, questioning the importance of including it. There are 24 pathogenic serogroups of *Leptospira*. It is not practical for most laboratories to test for all 24 serogroups, and it is probably not necessary. Further prevalence studies would allow the MAT to be focused on the serogroups that pose the greatest risk. Maintaining *Leptospira* cultures for use in the MAT is expensive due to the cost of media and maintaining the reference cultures [9]. To keep costs down, laboratories could test specific (or specialized) MAT panels based on relevance.

## 5. Conclusions

This study showed that animals were exposed to serovars not included in commercial bacterins in the U.S. and Puerto Rico, such as Bratislava (only in swine), Ballum, Hebdomadis, Tarassovi, and Szwajizak. The importance of these findings cannot be ignored. A prevalence study with a focus on isolation is greatly needed for the U.S. These data may suggest possible exposures, but since the MAT does not differentiate between natural exposure and vaccination, it is difficult to make solid conclusions. With isolation, the exact serogroup and serovar can be determined, and genotyping can be performed for more accurate epidemiology. In turn, MAT panels and bacterins can be developed to reflect the current prevalence of *Leptospira* in the U.S. and to ensure that positive samples are properly identified with the proper representative MAT panel.

## Figures and Tables

**Table 1:** MAT seroreactivity of the highest titer reactions against reference *Leptospira* antigens by the serum dilution. Samples with more than one highest titer reaction (76 samples) were not included.

	Serum Dilution									
Serogroup	1:100	1:200	1:400	1:800	1:1600	1:3200	1:6400	1:12,800	1:25,600	Total
Australis	24	16	4	2	1	0	0	0	0	47
Autumnalis	2	3	2	0	0	0	0	0	0	7
Ballum	20	7	2	2	1	0	0	0	0	32
Bataviae	0	0	0	0	0	0	0	0	0	0
Canicola	1	1	2	0	0	0	0	0	0	4
Grippotyphosa	5	6	12	8	3	1	1	0	1	37
Icterohaemorrhagiae	12	7	7	2	2	1	0	0	0	31
Pomona	4	6	4	4	0	0	0	0	1	19
Pyrogenes	3	1	0	0	0	0	0	0	0	4
Tarassovi	1	1	0	0	0	0	0	0	0	2
Sejroe	7	5	7	9	2	0	0	0	0	30
Hebdomadis	1	0	1	0	0	0	0	0	0	2
Szwajizak	2	0	0	1	0	0	0	0	0	3
Total	82	53	41	28	9	2	1	0	2	218
Total Percent Positive	37.6%	24.3%	18.8%	12.8%	4.1%	0.9%	0.5%	0%	0.9%	

## Data Availability

The data used to support this study are available from the corresponding author upon request.

## Supplementary Materials

The following supporting information can be downloaded at: https://www.mdpi.com/article/10.3390/tropicalmed8030183/s1, Table S1: MAT results against reference *Leptospira* antigens by species and localization, and Table S2: MAT seroreactivity against reference *Leptospira* antigens.
