# Supplementary material for: Animals Exposed to Leptospira Serogroups Not Included in Bacterins in the United States and Puerto Rico
Source: Trop Med Infect Dis. 2023 Mar 22;8(3):183. doi: 10.3390/tropicalmed8030183 (PMC10051158; doi:10.3390/tropicalmed8030183)
Supplement: Supplementary file 1 [file tropicalmed-08-00183-s001.zip › Supplementary Table S2 Anderson et al.pdf]

Table S2: Panel of antigens used in the microscopic agglutination test (MAT).

**Species**	**Serogroup**	**Serovar**	**Strain**
L. interrogans	Australis	Bratislava	Jez Bratislava
L. interrogans	Australis	Australis	Ballico
L. interrogans	Autumnalis	Autumnalis	Akiyami A
L. borgpetersenii	Ballum	Ballum	S 102
L. interrogans	Bataviae	Bataviae	Van Tienen
L. interrogans	Canicola	Canicola	H. Utrecht IV
L. interrogans	Grippotyphosa	Grippotyphosa	Andaman
L. interrogans	Hebdomadis	Hebdomadis	Hebdomadis
L. interrogans	Icterohaemorrhagiae	Copenhageni	M 20
L. interrogans	Mini	Szwajizak	Szwajizak
L. interrogans	Pomona	Pomona	Pomona
L. interrogans	Pyrogenes	Pyrogenes	Salinem
L. interrogans	Sejroe	Hardjo	Hardjoprajitno
L. borgpetersenii	Sejröe	Sejröe	M 84
L. borgpetersenii	Tarassovi	Tarassovi	Perepelitsin
